# A Stereolithography Appearance-Based Ultra-Wideband Wide-Beam Dielectric Resonator Antenna

**DOI:** 10.3390/s25226989

**Published:** 2025-11-15

**Authors:** Chenyang Song, Yubing Yuan, Shengbo Ye, Zihao Wang, Qunying Zhang, Xiaojun Liu, Guangyou Fang

**Affiliations:** 1 Key Laboratory of Electromagnetic Radiation and Sensing Technology, Aerospace Information Research Institute, Chinese Academy of Sciences, Beijing 100190, China; yuanyubing21@mails.ucas.ac.cn (Y.Y.); yesb@aircas.ac.cn (S.Y.); wangzihaowzh98@163.com (Z.W.); lxjdr@mail.ie.ac.cn (X.L.); gyfang@mail.ie.ac.cn (G.F.); 2 School of Electronic, Electrical, and Communication Engineering, University of Chinese Academy of Sciences, Beijing 100049, China

**Keywords:** dielectric resonator antenna (DRA), ultra-wideband (UWB), wide-beam, stereolithography (SLA), 3D printing

## Abstract

This paper presents a comprehensive study on the design, fabrication, and characterization of ultra-wideband (UWB) wide-beam dielectric resonator antennas (DRAs) using stereolithography (SLA)-based 3D printing technology. High-purity alumina ceramics were successfully fabricated through an optimized SLA process involving 80 wt.% solid loading and sintering. The proposed DRA design incorporates a vertical ground plane to achieve a compact footprint of 0.598λ_0_ × 0.491λ_0_ × 0.069λ_0_ (where λ_0_ is the wavelength corresponding to the center operating frequency of 4.15 GHz) while demonstrating an exceptional 70.59% relative bandwidth (2.75–5.75 GHz). A novel slot-loading technique was developed to correct radiation pattern distortions caused by higher-order modes, validated through both simulation and measurement. The antenna exhibits stable unidirectional radiation patterns with a wide half-power beamwidth in both the E-plane and H-plane and a gain of 2.5–5.5 dB across the operating band. This work establishes SLA as a viable manufacturing approach for high-performance RF components.

## 1. Introduction

Modern wireless systems are driving societal digital transformation at an unprecedented pace. With the rapid development of next-generation radar systems, mobile communications, the Internet of Things (IoT), and other technologies, the performance requirements for antenna systems have undergone multidimensional leaps: not only is a wider impedance bandwidth needed to support high-speed data transmission, but stable radiation patterns, compact physical size, and good compatibility with manufacturing processes are also essential [1,2,3,4]. Among these technical demands, achieving a balance between ultra-wideband performance and stable radiation characteristics remains a core challenge in the field of antenna design.

As an efficient radiating structure, dielectric resonator antennas (DRAs) have attracted widespread attention in recent years due to their advantages such as low loss, high radiation efficiency, flexible mode excitation, and a high degree of design freedom [5]. Compared to traditional microstrip patch antennas, DRAs avoid surface wave loss and exhibit higher radiation efficiency; compared to metallic antennas, their size can be adjusted via the dielectric constant, offering designers greater flexibility [6]. However, since DRAs are frequency-dependent antennas based on resonant modes, the bandwidth limitation of conventional DRAs remains significant. Early studies achieved an impedance bandwidth of 25% by stacking multiple resonators [7] or employed composite or special geometric structures (such as conical, stepped, well-shaped, or tetrahedral designs) to extend bandwidth [8,9,10,11]. Additionally, introducing an air gap between the DRA and the ground plane can further enhance bandwidth [12]. Multilayer and loaded DRAs can also effectively broaden bandwidth, and loading monopoles with suspended annular dielectric resonators has been demonstrated to achieve bandwidth expansion [13,14]. However, these methods often come at the cost of structural complexity or pattern stability, which directly affects the practical value of DRAs. In recent years, novel feeding techniques and hybrid-mode excitation schemes have been proposed, further advancing the performance of DRAs in terms of bandwidth [15,16,17]. Nevertheless, the bandwidth of existing DRAs mostly ranges from about 25% to 60%, and many of these antennas exhibit bipolar, omnidirectional, or irregular radiation patterns [18,19]. This indicates that developing practical ultra-wideband DRAs with both broadband performance and unidirectional wide-beam characteristics that meet the requirements of modern wireless applications such as radar and communication base stations remains a major bottleneck in current research.

Moreover, despite continuous innovation in design methods, traditional manufacturing processes have become a bottleneck restricting the development of DRAs, particularly for those requiring complex geometries or customized dielectric properties. Subtractive methods like precision machining and powder sintering struggle to fabricate intricate three-dimensional structures efficiently, often resulting in significant material waste and high cost. The emergence of additive manufacturing (3D printing) technology offers a promising alternative. However, prevalent techniques such as selective laser sintering (SLS) and fused deposition modeling (FDM) present notable limitations for high-frequency applications. SLS-printed ceramic parts often exhibit low microscopic density and high internal porosity due to insufficient powder melting, leading to undesirable fluctuations in the dielectric constant and increased loss [20,21,22]. Similarly, FDM technology is constrained by nozzle diameter and interlayer bonding, which produces visible layer lines and surface roughness that can disrupt electromagnetic field distribution and limit performance at higher frequencies [23].

In contrast, stereolithography (SLA) stands out as a superior solution for manufacturing high-performance ceramic DRAs. SLA utilizes area projection of ultraviolet light to cure liquid photosensitive ceramic slurry, enabling micron-level resolution, excellent surface quality, and nearly dense ceramic microstructures. This technical capability ensures superior stability and consistency in dielectric properties, which is critical for RF applications. For instance, previous studies have demonstrated that SLA-fabricated alumina-based DRAs exhibit favorable radiation characteristics and achieve broadband impedance matching [24,25]. These advantages establish SLA as a viable manufacturing approach that not only overcomes the limitations of traditional methods but also enables the realization of complex electromagnetic structures previously unattainable, thereby creating new pathways for designing next-generation high-performance dielectric resonator antennas. In summary, the performance of DRAs is constrained by multiple factors, including design freedom and bandwidth. Against this research background, this paper proposes a novel ultra-wideband wide-beam dielectric resonator antenna based on stereolithography technology. By innovatively combining the SLA fabrication process of high-purity alumina ceramic with a vertical ground structure design, ultra-wideband characteristics are achieved in a compact size. Simultaneously, electromagnetic field control techniques address radiation pattern distortion across a wide operating band, enabling wide-beam radiation in both the E-plane and H-plane. This work holds significant importance for expanding the coverage of modern radar, communication, and IoT technologies, offering a complete solution from material preparation to antenna design. It provides a new technical path for high-performance antenna design in future communication systems and pushes the boundaries of additive manufacturing in the RF field.

In the following sections, Section 2 describes the SLA-based dielectric ceramic fabrication and characterization. The design, simulation, and measurement of SLA-fabricated UWB wide-beam DRAs is studied in Section 3. The radiation pattern correction method and results are given in Section 4. In Section 5, conclusions are drawn.

## 2. Preparation and Characterization of Dielectric Ceramics via SLA

### 2.1. SLA-Fabricated Dielectric Ceramics

Dielectric resonator antennas operate through electromagnetic wave resonance within dielectric structures. When the physical dimensions of the resonator match the wavelength of stored electromagnetic waves, strong resonant modes emerge that can be effectively radiated through proper feeding mechanisms. The preparation and characterization of dielectric materials therefore constitute fundamental prerequisites for DRA design.

**Figure 1:**
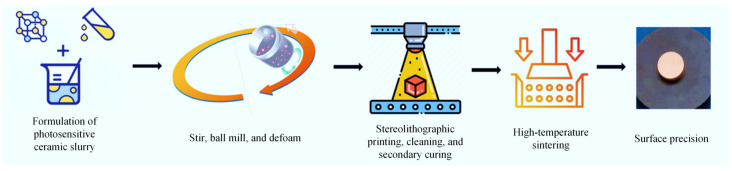
Process of SLA-based ceramic fabrication.

As illustrated in Figure 1, the SLA-based ceramic fabrication process comprises five critical steps:

(1) Formulation of photosensitive ceramic slurry through mixing nano-ceramic powder with photosensitive dispersants and stabilizers.
(2) Mixing nanoalumina ceramic powder, photosensitive resin, photo initiator, and dispersant with the ball mill, stir, and defoam method.
(3) Stereolithographic printing with subsequent cleaning and secondary curing processes.
(4) High-temperature debinding and sintering in a controlled-atmosphere furnace [26].
(5) Precision surface finishing to produce final SLA dielectric ceramics.

The selection of ceramic powder fundamentally determines the physical properties of the resulting dielectric ceramics. For DRA applications, the dielectric characteristics—specifically the relative permittivity (ε_r_) and loss tangent (tan δ)—represent the most critical parameters. Excessive permittivity increases the antenna Q-factor, thereby reducing operational bandwidth, while insufficient permittivity necessitates larger antenna volumes. Elevated dielectric losses compromise radiation efficiency and gain, negating the inherent advantages of DRAs. This is because a high loss tangent (tan δ) signifies the conversion of a portion of the radiated electromagnetic energy into heat within the dielectric material. This energy dissipation directly reduces the radiation efficiency (η) and consequently lowers the realized gain (G), since G ∝ η × D, where D is the directivity. This work utilizes α-alumina (α-Al_2_O_3_) with a trigonal crystal structure as the ceramic material, exhibiting a typical relative permittivity of 8–11 that optimally balances bandwidth and volume requirements [27]. Particle size distribution represents another crucial parameter, significantly influencing both slurry rheology and final sintered properties [28]. While finer particles, which possess a higher specific surface area, increase slurry viscosity and agglomeration tendency—potentially impairing self-leveling during printing—excessively large particles produce microstructural porosity that degrades dielectric and mechanical properties after sintering [26]. Through systematic evaluation, the alumina ceramic powder is formed by mixing alumina powder with a particle size of 300 nm and 500 nm according to a mass ratio of 3:1, and adding an appropriate amount of large-particle-size powder can effectively reduce the number of alumina particles in a unit volume, reduce the interaction between particles, and reduce the viscosity of ceramic slurry.

Dispersants were incorporated to address the challenges of particle agglomeration and viscosity control. The hydroxyl-rich surfaces of Al_2_O_3_ particles promote electrostatic attraction through van der Waals forces, necessitating dispersants to achieve homogeneous slurry formulations suitable for precision printing. The resin system employs 1,6-hexanediol diacrylate (HDDA) as the primary monomer, selected for its optimal combination of low viscosity (beneficial for slurry formulation) and excellent photocuring characteristics. Table 1 summarizes the properties of various photocurable resins evaluated during formulation development. The resin, photoinitiator and dispersant are 1,6-hexanediol diacrylate (HDDA), trimethyl benzoyl-diphenyl phosphine oxide (TPO) and propylene glycol methyl ether acetate (PGMEA), respectively. The slurry preparation process is to mix alumina powder (65 wt.%), resin (32 wt.%), dispersant (1 wt.%) and photoinitiator (2 wt.%) and then add it to the star mill for ball milling for 4 h. The slurry is cured by 405 nm ultraviolet light after stirring and defoaming, with a single-layer exposure energy of 100 mJ/cm^2^ and a printing layer thickness of 50 μm. After printing, it is ultrasonic cleaned with 95% concentration alcohol, post-cured with the same wavelength light, and degreased and densified by sintering at 600 °C (heating rate 2 °C/min, holding time 2 h) and sintering at 1600 °C (heating rate 2 °C/min, holding time 3 h), respectively, finally forming ceramic medium blocks.

**Figure 2:**
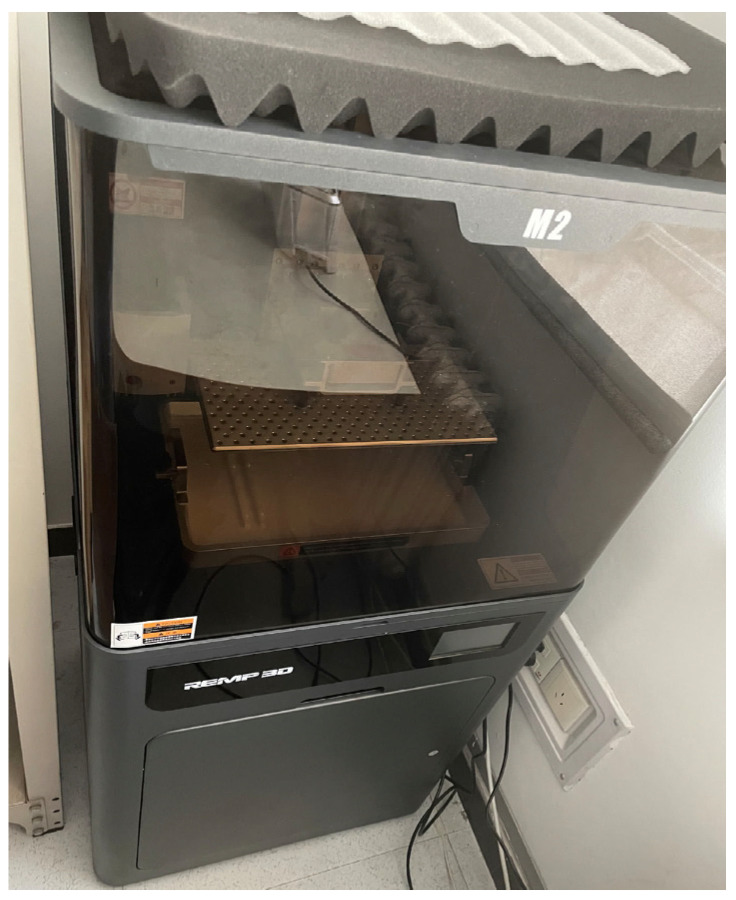
Commercial SLA system.

Following powder and resin preparations, the components were homogenized through ultrasonic dispersion, mechanical mixing, and ball milling to produce the final ceramic slurry. As shown in Figure 2, the printing process utilized a commercial SLA system (REMP M2, Manufactuer: REMP Information Technology Co., Ltd., Nanjing, China) featuring a 10 μm layer thickness and 18 μm^2^ XY-plane resolution, enabling fabrication of high-precision dielectric structures unattainable through subtractive methods.

**Figure 3:**
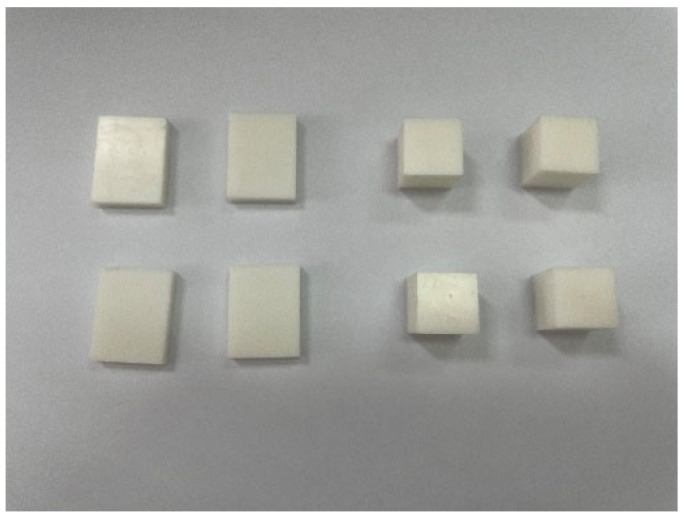
Prototyped SLA-fabricated dielectric ceramics.

After post-processing, including ultrasonic cleaning in 99% alcohol, UV post-curing, and high-temperature sintering, representative sintered samples were fabricated, as shown in Figure 3.

### 2.2. Characterization of SLA-Fabricated Dielectric Ceramics

**Figure 4:**
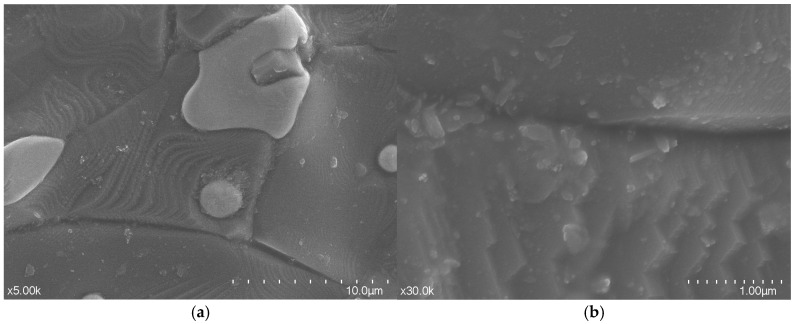
SEM images of sintered alumina flakes: (**a**) low-magnification view showing overall morphology (×5000, scale bar: 10 μm); (**b**) high-resolution image revealing nanoscale grain boundaries (×30,000, scale bar: 1 μm).

For the purpose of validating the physical and dielectric properties of the fabricated ceramics, microstructural analysis is conducted via scanning electron microscopy (SEM). The results are shown in Figure 4. Figure 4a shows the low-magnification view showing overall morphology. The picture shows that the particles exhibit irregular polygons, which conform to the grain characteristics of typical sintered alumina, and the samples are well prepared with high edge definition. Figure 4b shows the high-resolution image, revealing nanoscale grain boundaries. According to the picture, clear polygonal grains with straight and sharp grain boundaries indicate that the grain growth during the sintering process is sufficient, the overall density is good, and only nanoscale pores are locally present.

**Figure 5:**
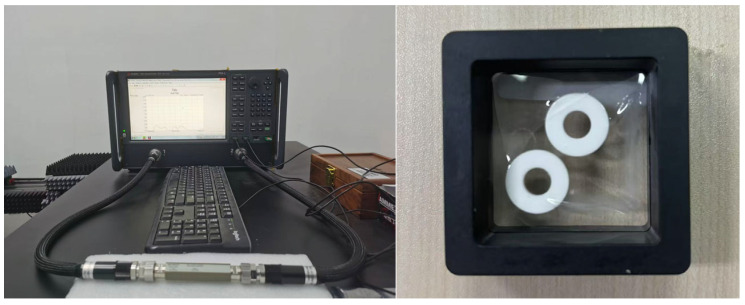
Coaxial method dielectric property measurement setup and the sample under test.

For dielectric property characterization, this study implemented a resonant cavity approach, a coaxial approach, and full-wave DRA simulations for dielectric constant and loss measurements. First, the resonant cavity approach was conducted. The fundamental relationship for a rectangular cavity (*a* × *b* × *c*) completely filled with a dielectric material of relative permittivity ε_r_ is given by [30]

$$f_{r,mnp}=\frac{c_{0}}{2\pi \sqrt{\varepsilon_{r}}}\sqrt{{\left(m\pi /a\right)}^{2}+{\left(n\pi /b\right)}^{2}+{\left(p\pi /c\right)}^{2}}$$

where *m*, *n*, and *p* represent the modal integers corresponding to the spatial oscillation periods along each dimensional axis, while *c*_0_ denotes the speed of light in a vacuum. Consequently, under a constant cavity geometry, the resonant frequency of each mode exhibits an inverse proportionality to the square root of the relative permittivity of the filling material. The resonant frequencies are identified through impedance peaks in the cavity’s input response. The resonant cavity configuration employed for ceramic sample characterization is illustrated in Figure 5. When the dielectric material partially fills the cavity along its shortest dimension (designated as *c*), the modified dispersion relation governing the resonant frequency ω for fundamental modes must satisfy the following relationship:

$$\frac{\beta_{y0}}{\varepsilon_{0}}\tan\left(\beta_{y0}\left(c-h\right)\right)=-\frac{\beta_{yd}}{\varepsilon_{r}\varepsilon_{0}}\tan\left(\beta_{yd}h\right)$$

$$\beta_{y0}=\sqrt{\omega^{2}\mu_{0}\varepsilon_{0}-{\left(m\pi /a\right)}^{2}+{\left(n\pi /b\right)}^{2}}$$

$$\beta_{yd}=\sqrt{\omega^{2}\mu_{0}{\varepsilon_{r}\varepsilon }_{0}-{\left(m\pi /a\right)}^{2}+{\left(n\pi /b\right)}^{2}}$$

where *h* is the height of the dielectric filling. Notably, even minimal air gaps between the cavity walls and dielectric substrate can induce measurable resonant frequency shifts. To ensure measurement accuracy, dimensional characterization of test specimens was performed using vernier calipers, enabling subsequent determination of the unknown relative permittivity through inverse calculation.

Regarding loss tangent characterization, under the assumption of negligible coupling losses, the quality factor *Q* of the resonant cavity can be expressed as the reciprocal sum of ohmic and dielectric losses [30,31]:

$$Q^{-1}=\frac{R_{s}}{G}+\tan\left(\delta_{e}\right)$$

where *R_s_* represents the surface resistance of the metallic cavity walls and tan(*δ_e_*) denotes the loss tangent of the dielectric filling. *G* is the geometric factor with a unit of Ohm, which characterizes the relationship between the stored magnetic energy in the resonator and the power dissipated on the conducting surfaces. It relates the surface resistance to the ohmic loss contribution, where a higher *G* implies a lower ohmic loss for a given *R_s_*. The dielectric loss tangent was extracted by subtracting the calculated geometric factor and surface resistance contributions from the measured quality factor. Through the measurements, the relative permittivity and loss tangent are 9.3 and 1.1 × 10^−3^ at 3 GHz.

As shown in Figure 4, the Keysight N5222B Performance Network Analyzer (PNA) and its coaxial dielectric measurements kit were used. The complex permittivity was further characterized using the coaxial transmission line method [32,33,34]. A toroidal-shaped sample with an inner diameter of 3 mm, an outer diameter of 7 mm, and a thickness of 2 mm was precisely fabricated to match the commercial 7mm coaxial test fixture. The S-parameters of the sample-loaded fixture were measured, and the dielectric properties were extracted. The ceramic under test is an SLA-fabricated ring ceramic with a 3 mm inner diameter, 7 mm outer diameter, and 2mm height. The results show that the relative permittivity and loss tangent are 9.2 and 1.6 × 10^−3^ at 3 GHz, which agree well with the previous results.

It is noteworthy that the measured loss tangent (on the order of 10^−3^) is higher than the theoretical value for fully dense, high-purity polycrystalline Al_2_O_3_ (typically ~10^−4^). This is a common characteristic of 3D-printed ceramics, primarily attributed to residual nanoscale porosity and potential impurities introduced during the slurry preparation and sintering processes, as observed in the SEM image (Figure 4b). Despite this, the achieved loss tangent is sufficiently low for high-performance DRA applications and is consistent with values reported for other SLA-fabricated functional ceramics in the RF domain.

**Figure 6:**
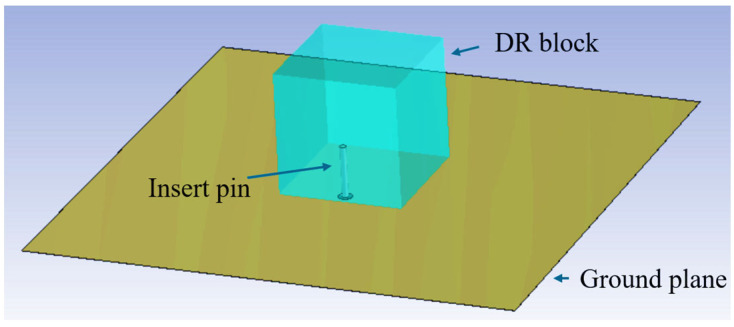
Simulation model of inset-fed DRA.

**Figure 7:**
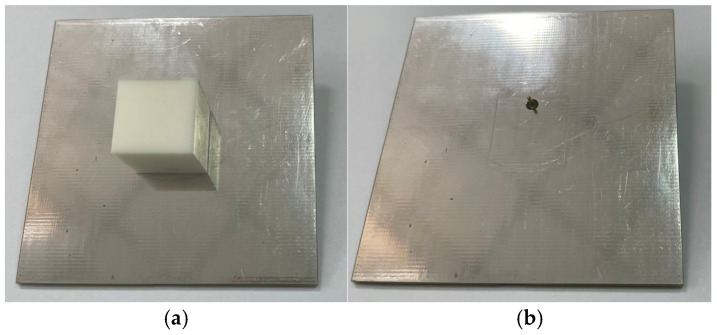
Validation of inset-fed DRA: (**a**) top view and (**b**) bottom view.

To further validate the dielectric constant of the SLA-fabricated dielectric ceramics, a probe-fed DRA is designed, simulated, and fabricated. The designed DRA is a cube with a length, width, and height of 16.6 mm, and the probe is inserted 6.9 mm into the center of one side to feed the antenna, which is shown in Figure 6 and Figure 7. According to the DRA field distribution theory, the resonant frequency of the TE_ymnl_ mode of the rectangular DRA can be expressed as [35]

$$f_{mnl}=\frac{c}{2\pi \sqrt{\varepsilon_{r}}}\sqrt{{k_{x}}^{2}+{k_{y}}^{2}{{+k}_{z}}^{2}}$$

where

$$k_{x}=\frac{m\pi }{a}$$

$$k_{y}\tan\left(\frac{k_{y}b}{2}\right)=\sqrt{\left(\varepsilon_{r}-1\right){k_{0}}^{2}-{k_{y}}^{2}}$$

$$k_{z}=\frac{l\pi }{2d}$$

**Figure 8:**
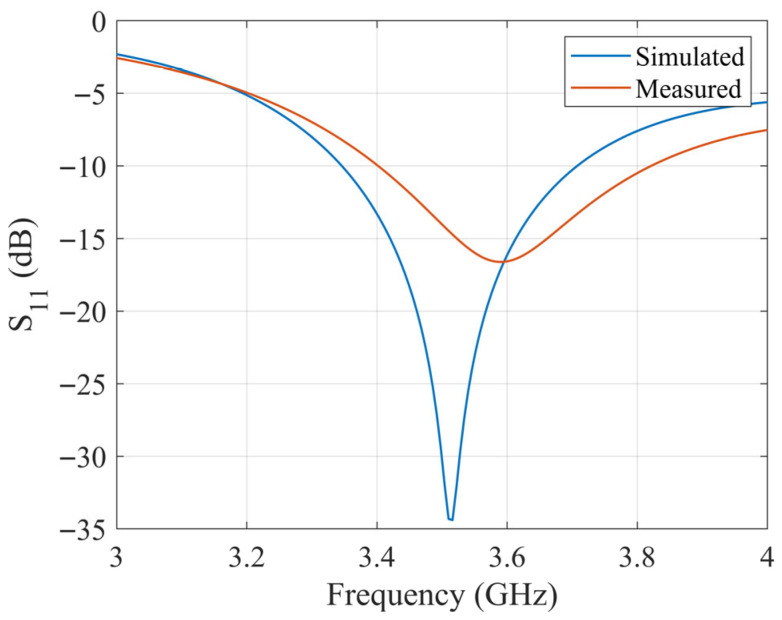
Simulated and measured S_11_ of inset-fed DRA.

*a*, *b*, and *d* are the lengths of the DRA in the *x*, *y*, and *z* directions, respectively, and *k* is the wavenumber. According to the theory and the design, the resonant frequency of the excited TE_y111_ mode is about 3.5 GHz. The S_11_ of the designed antenna is simulated with CST 2024 full-wave electromagnetic simulation software and measured with a Keysight E5063A vector network analyzer (VNA) in the anechoic chamber. The simulated and measured S_11_ results are shown in Figure 8. According to the results, the simulation and measurement agree well, which proves the accuracy of the previous dielectric characteristics measured.

## 3. Design of SLA-Based UWB Wide-Beam DRA

### 3.1. SLA-Fabricated UWB DRA

**Figure 9:**
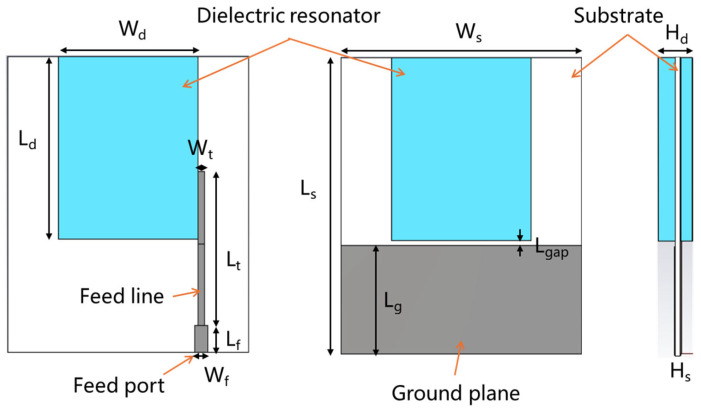
Dimensions of the proposed UWB wide-beam DRA.

Figure 9 shows the designed ultra-wideband wide-beam DRA model with a vertical reference ground. This antenna is derived from a metal-strip-coupled DRA with a horizontal reference ground. In comparison, the proposed antenna features a smaller ground plane, significantly reducing the overall size (for conventional DRAs with a horizontal ground, the ground plane typically exceeds four times the length of the dielectric resonator). Additionally, the modified boundary conditions at the bottom of the dielectric ceramic (along the z-axis/radiation direction) introduce new resonant modes. Furthermore, a gap is introduced between the dielectric resonator and the edge of the ground plane to enhance the electric field on the opposite side of the feed probe and provide better balance between the two sides, thereby mitigating radiation pattern distortion caused by offset feeding.

The antenna also incorporates a robust mechanical fixation structure, eliminating the common drawbacks of traditional horizontally grounded DRAs, such as difficult adjustment, fixation, and feeding. The antenna consists of a dielectric substrate, an embedded dielectric ceramic, a feed line, and a ground plane. The feed line section includes an impedance matching network to achieve a 50-Ohm input impedance.

### 3.2. Paramatric Study

**Figure 10:**
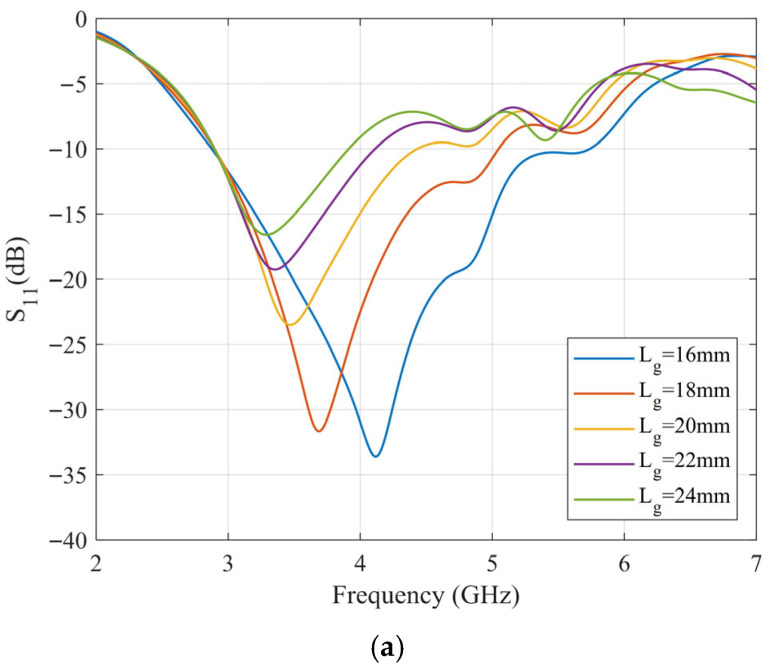

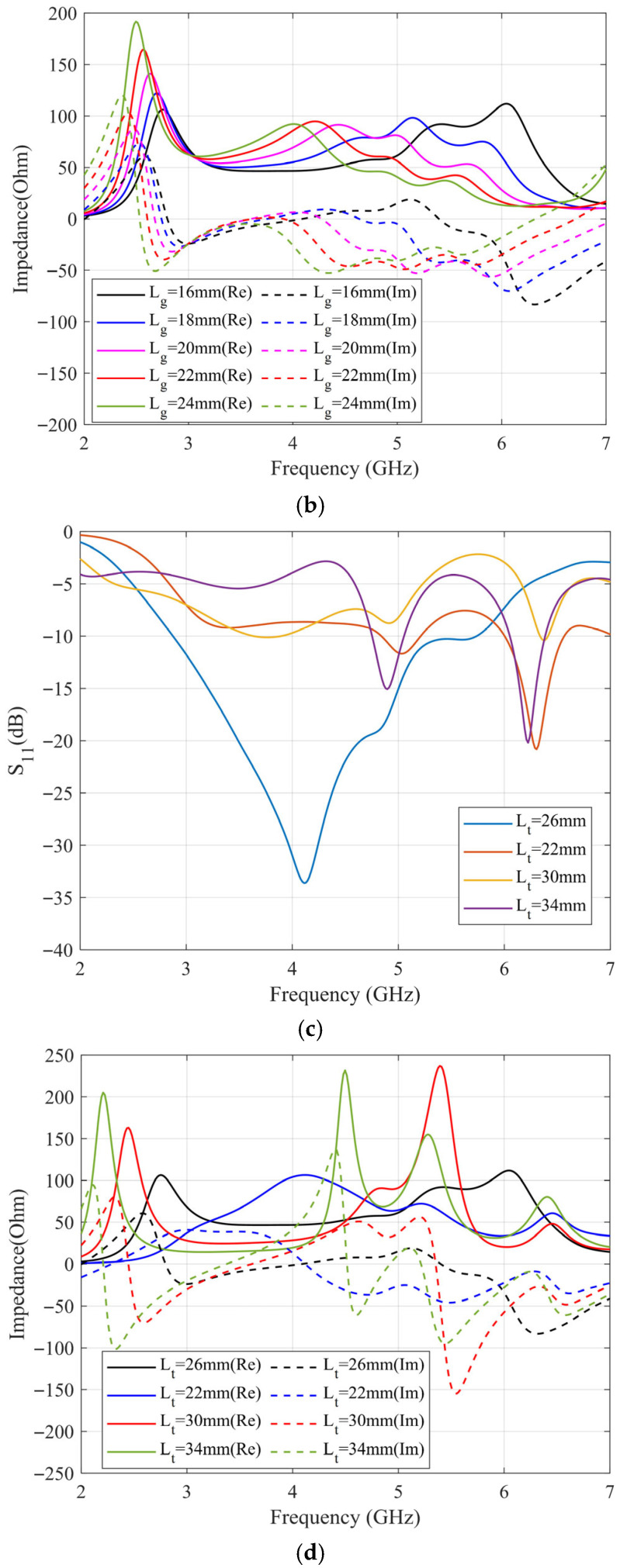

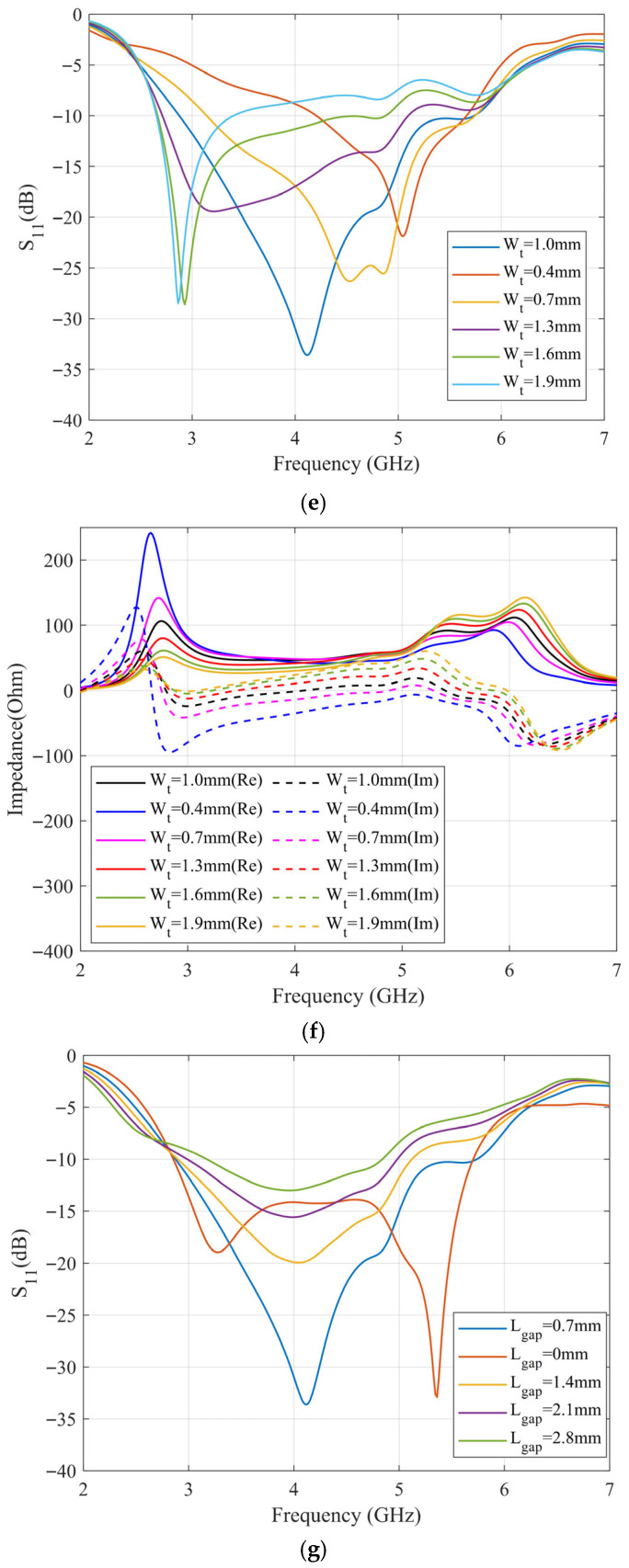

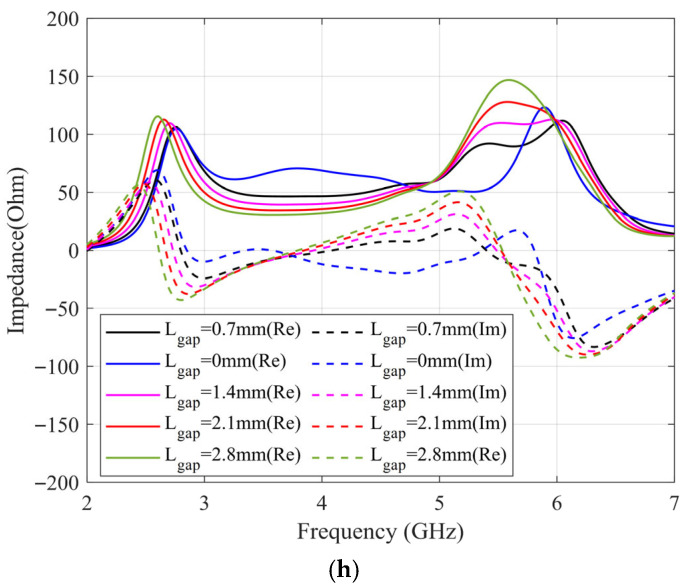
(**a**) Effect of vertical ground plane length L_g_ on resonant frequency, (**b**) effect of vertical ground plane length L_g_ on impedance, (**c**) effect of matching feeder length L_t_ on resonant frequency, (**d**) effect of matching feeder length L_t_ on impedance, (**e**) effect of matching feeder wire width W_t_ on resonant frequency, (**f**) effect of matching feeder wire width W_t_ on impedance, (**g**) effect of slit width L_gap_ on resonant frequency, (**h**) effect of gap width L_gap_ on impedance.

For the proposed DRA, although the influence of dielectric antenna dimensions on resonant modes and impedance has been extensively studied, the presented structure is inherently an unbalanced design. The grounding effect at the edge of the vertical ground plane causes the antenna’s performance to be significantly affected by the size of the ground plane and the gap width between the ground plane and the radiator. Additionally, the length of the ground plane, as well as the length and width of the feedline (which serves as an impedance-matching transformer at the feed port), critically influence the DRA’s performance. To analyze these parameters, a parametric study is conducted using the CST full-wave electromagnetic simulation software. In each simulation, only the parameter under investigation was varied, while all other geometric parameters were held constant at their optimized values listed in Table 2. The antenna employs a Rogers RO6002 substrate (thickness: 0.762 mm, ε_r_ = 2.94, tanδ = 0.0012). Figure 10 shows the effect of varying these key parameters on the antenna’s input impedance.

According to the results, the length of the vertical ground plane and the matching feedline critically influence the antenna’s performance by modifying the external coupling to the DRA, which in turn shifts the resonant frequency. In contrast, the width of the matching feedline and the gap width primarily affect the imaginary part of the antenna’s input impedance, fine-tuning the matching condition. After optimization, the antenna parameters are listed in Table 2. The antenna has compact dimensions of only 0.598λ_0_ × 0.491λ_0_ × 0.069λ_0_ (where λ_0_ is the wavelength corresponding to the center operating frequency of 4.15 GHz).

### 3.3. Simulation and Measurement

**Figure 11:**
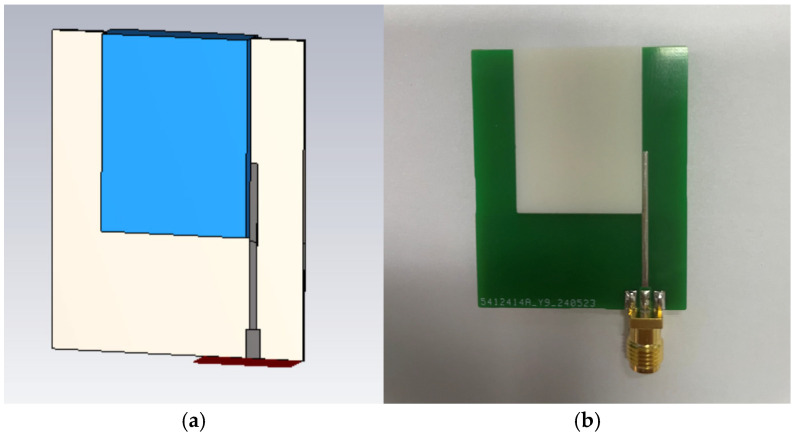
(**a**), Simulated UWB wide-beam DRA, (**b**) prototyped UWB wide-beam DRA.

**Figure 12:**
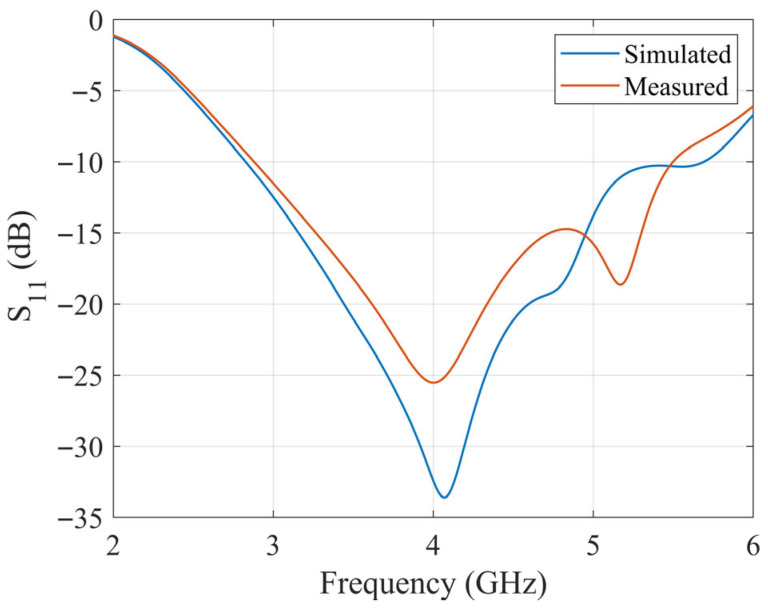
Simulated and measured S_11_ of the proposed UWB wide-beam DRA.

**Figure 13:**
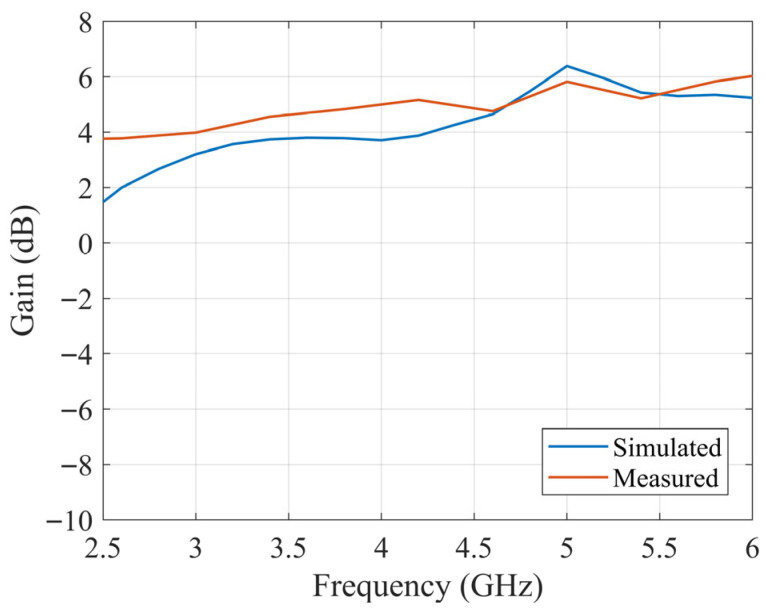
Simulated and measured gain over frequency of the proposed UWB wide-beam DRA.

According to the optimized dimensions, the designed antenna is simulated with CST software. The ceramic block is fabricated with SLA, and the substrate is fabricated with a printed circuit board (PCB). The simulated and fabricated antennas are shown in Figure 11. The prototyped antenna is measured with a Keysight E5063A vector network analyzer in an anechoic chamber. During the measurement, a calibrated horn antenna with known gain is set in the far-field of the antenna under test to conduct the gain and radiation pattern measurements. Figure 12 shows the simulated and measured S_11_ of the proposed antenna. The antenna achieves a 65% bandwidth (2.80–5.50 GHz) via multi-mode excitation, with experimental results matching simulations. The simulated and measured gain are shown in Figure 13. The simulated and measured results agree well. According to the measurements, the antenna has a peak gain of 5.8 dB with a gain variation smaller than 2 dB.

**Figure 14:**
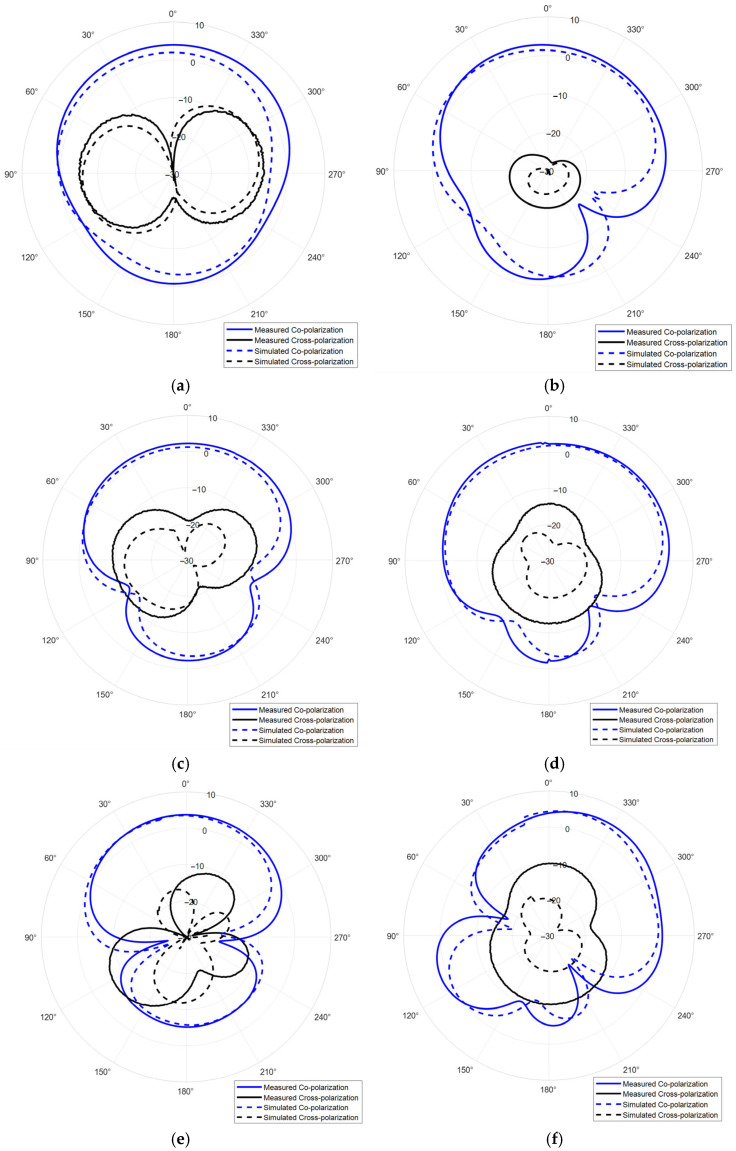
Simulated and measured radiation patterns (gain in dB) of the proposed UWB wide-beam antenna, (**a**) H-plane at 3.5 GHz, (**b**) E-plane at 3.5 GHz, (**c**) H-plane at 4.5 GHz, (**d**) E-plane at 4.5 GHz, (**e**) H-plane at 5.5 GHz, (**f**) E-plane at 5.5 GHz.

The simulated radiation patterns at 3.5 GHz, 4.5 GHz, and 5.5 GHz are shown in Figure 14. According to the results, the antenna has a wide beamwidth, which can significantly improve the coverage of radar and communication systems. However, due to utilization of a high-order resonant mode and single-side feed, the antenna has obvious radiation pattern distortion in its high frequency. Therefore, the DRA radiation pattern correction approach is studied.

## 4. UWB Wide-Beam DRA Radiation Pattern Correction

**Figure 15:**
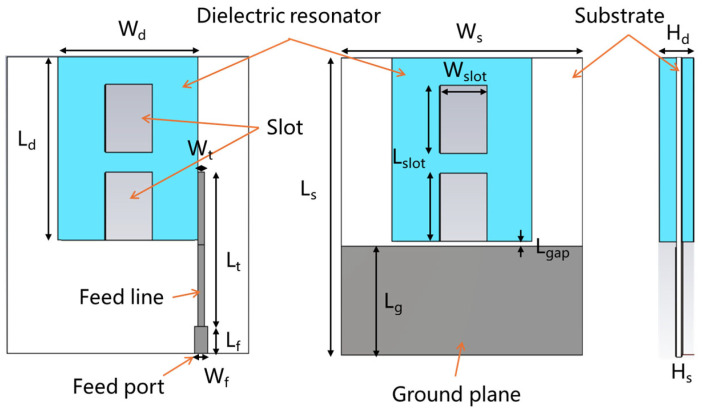
Dimensions of slotted UWB wide-beam DRA.

Figure 14e reveals significant pattern distortion in the upper operating frequency band, manifesting as asymmetric radiation and elevated sidelobes in the main beam direction. This phenomenon is attributed to the excitation of hybrid higher-order resonant modes. Although we have introduced a gap between the dielectric resonator and ground plane edge to enhance electric field balance on both sides of the feed probe, the persistence of higher-order modes still induces substantial radiation pattern degradation. To address this issue, this study proposes an ultra-wideband DRA pattern correction technique. By analyzing the near-field distribution within the resonator at distortion-prone frequencies, a slotted dielectric resonator was fabricated via additive manufacturing. The slot is strategically positioned in the central region of disordered high-order field concentration. Given the resonator’s thin profile, the slot minimally perturbs desired radiation modes while effectively displacing disordered higher-order fields beyond the operational band, thereby improving field symmetry and reducing antenna weight. Figure 15 presents the optimized ultra-wideband DRA, with slots placed at the electric field hotspot to enhance balance without compromising performance. Refined parameters are summarized in Table 3.

**Figure 16:**
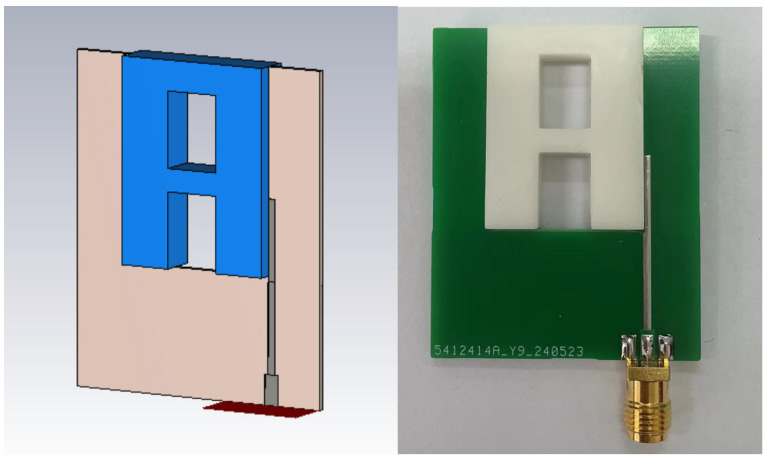
Simulated and prototyped slotted UWB wide-beam DRA.

**Figure 17:**
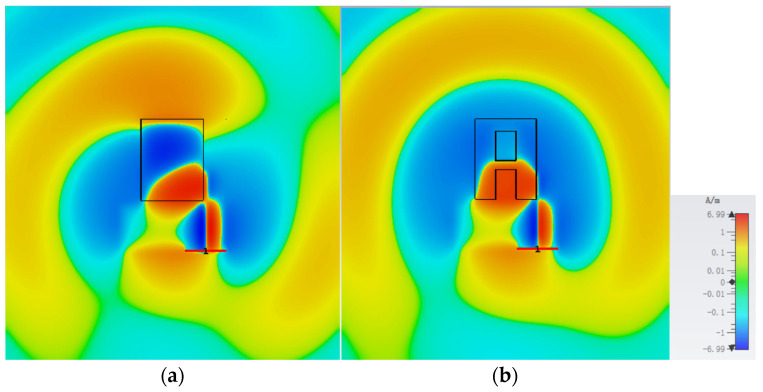
Simulated H-field distribution of (**a**) original and (**b**) slotted UWB wide-beam DRA.

Similarly, the antenna is simulated with CST full-wave electromagnetic simulation software and fabricated with SLA. The simulated and measured DRAs are shown in Figure 16. Figure 17 shows the H-field distributions of the original antenna and the slotted antenna at 5.5 GHz. The results demonstrate that the introduced slot effectively improves field symmetry, which leads to the correction of radiation patterns.

**Figure 18:**
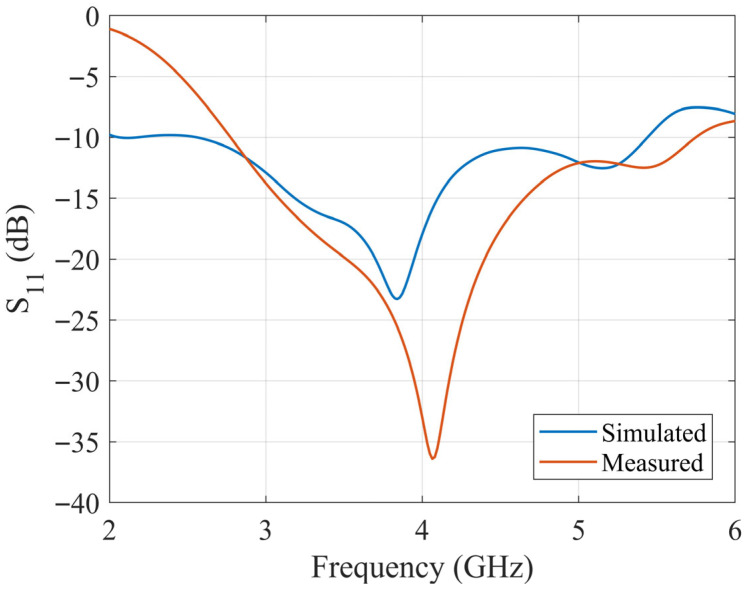
Simulated and measured S_11_ of slotted UWB wide-beam DRA.

**Figure 19:**
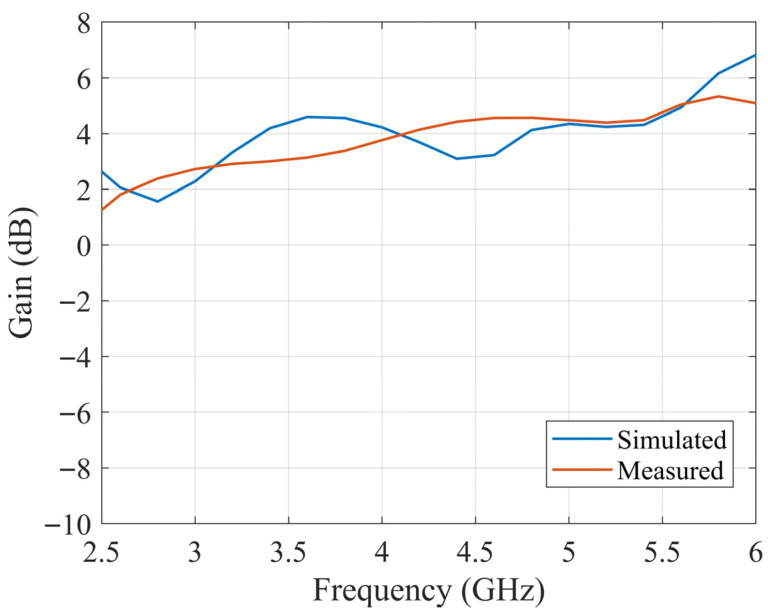
Simulated and measured gain over frequency of slotted UWB wide-beam DRA.

**Figure 20:**
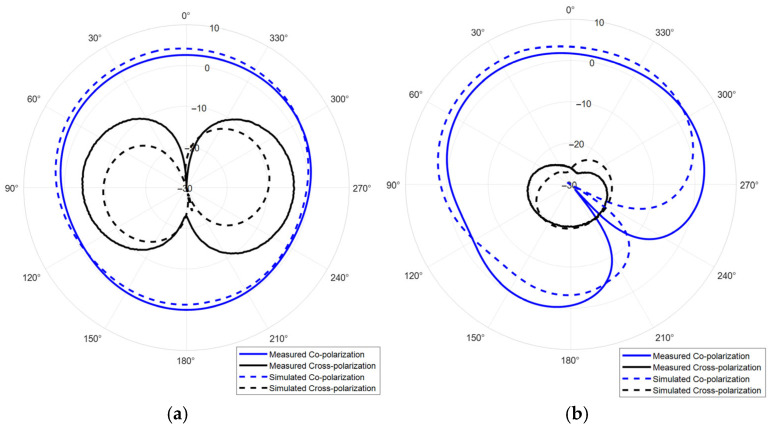

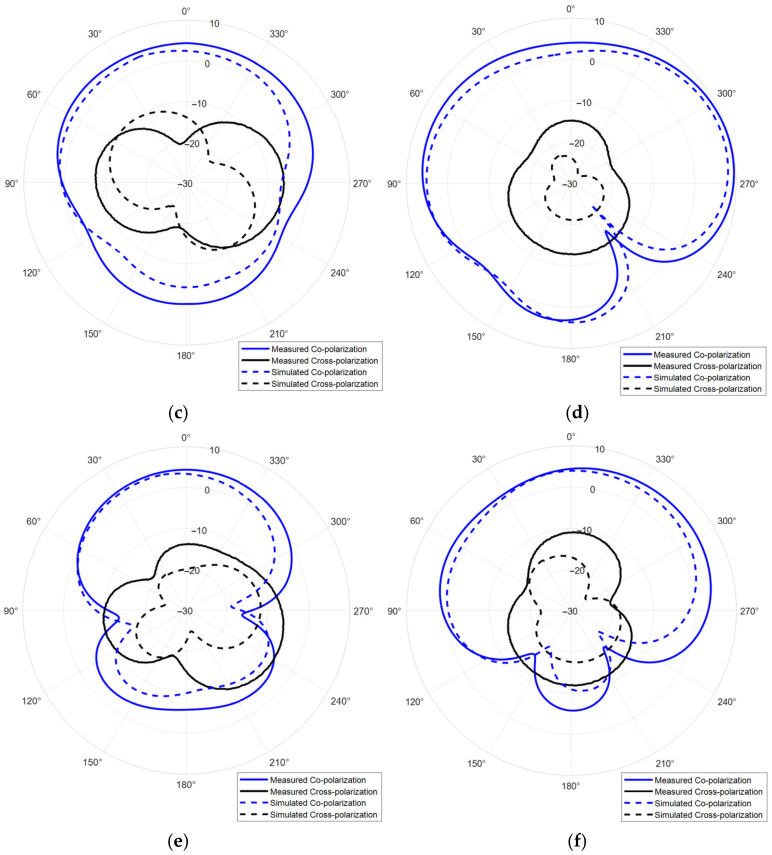
Simulated and measured radiation patterns (gain in dB) of the slotted UWB wide-beam antenna, (**a**) H-plane at 3.5 GHz, (**b**) E-plane at 3.5 GHz, (**c**) H-plane at 4.5 GHz, (**d**) E-plane at 4.5 GHz, (**e**) H-plane at 5.5 GHz, (**f**) E-plane at 5.5 GHz.

**Figure 21:**
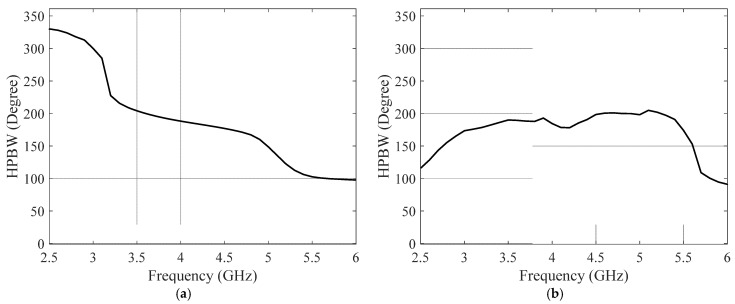
HPBW over frequency of the slotted UWB wide-beam antenna on (**a**) H-plane and (**b**) E-plane.

The slotted DRA is measured with a Keysight E5063A vector network analyzer in the anechoic chamber. During the measurement, a calibrated horn antenna with known gain is set in the far-field of the antenna under test to conduct the gain and radiation pattern measurements. Figure 18 shows the simulated and measured S_11_ of the proposed antenna. The simulated and measured results agree well. The measured S_11_ shows an −10 dB impedance bandwidth of 2.75–5.75 GHz (70.59%), which is improved comparing to the original design. Figure 19 shows the simulated and measured gain of the proposed antenna, which are in good agreement over the entire band. The minor discrepancy observed around 4.5 GHz is attributed to cumulative experimental tolerances and material losses in the simulation and does not affect the overall performance validation. A 5.5 dB peak gain with less than 3 dB gain variation is achieved. Figure 20 and Figure 21 show the measured radiation pattern at different frequencies and half-power beamwidth (HPBW), respectively. According to the results, a unidirectional UWB wide-beam antenna is achieved.

Table 4 summarizes the performance comparison between the proposed antenna and several state-of-the-art dielectric resonator antennas. The results demonstrate that our SLA-fabricated DRA exhibits a remarkable balance among multiple key performance metrics. Most notably, it achieves the widest fractional bandwidth (70.59%) while maintaining a compact electrical size. Furthermore, the antenna displays significantly wider half-power beamwidth in both the E- and H-planes compared to other designs, underscoring its exceptional wide-beam characteristic. This combination of ultra-wideband operation, compact footprint, and stable wide-beam radiation distinguishes our work from existing approaches and highlights its suitability for applications requiring broad angular coverage and miniaturization, such as base station and through-wall radar.

## 5. Conclusions

This manuscript has presented the comprehensive design, fabrication, and experimental validation of a stereolithography-based UWB wide-beam DRA. The work demonstrates the successful integration of advanced additive manufacturing techniques with high-performance RF component design, offering a viable pathway to overcome the limitations of traditional subtractive manufacturing methods for complex dielectric structures and high-precision rapid manufacturing.

One of the core accomplishment is the development of an optimized SLA process that yields high-purity alumina ceramics with excellent RF properties (ε_r_ ≈ 9.3, tan δ ≈ 1.1 × 10^−3^), enabling the fabrication of a novel DRA. Furthermore, the antenna achieves a compact size with a 70.59% measured operation bandwidth (2.75–5.75 GHz) through multi-mode excitation. Crucially, an innovative slot-loading technique was introduced to effectively correct higher-order mode-induced pattern distortion by optimizing the electromagnetic field distribution. By strategically placing a slot in the region of disordered field concentration, the field symmetry was significantly improved, resulting in stable unidirectional radiation and a wide beamwidth across the entire band, with a consistent gain of 2.5–5.5 dB. The antenna shows UWB unidirectional wide-beam radiation on both the E- and H-planes and can easily be configured to other frequency bands, with advantages for radar, Internet of Things, and communication applications, such as through-wall radar, microwave monitoring, and communication base stations.

In conclusion, this work proposes an SLA-based DRA design, with its compact size, ultra-wide bandwidth, stable gain, and corrected radiation patterns, presenting a strong candidate for modern communication, radar, and Internet of Things applications. Moreover, SLA is established as a highly capable manufacturing technology for producing high-performance, geometrically complex dielectric resonators that are valuable for next-generation wireless systems.

## Figures and Tables

**Table 1:** Properties of commonly used resins [29].

Index	Functionality	Molar Mass/(g·mol^−1^)	Density/(g·cm^−3^)	Viscosity/(mPa·s)	Refractive Index
AM	1	71	1.32	Solid	19.230
2HEA	1	116	1.01	8~10	1.445~1.450
HEMA	1	130	1.07	6~11	1.453
4HBA	1	144	1.04	10~25	1.452~1.454
St	1	104	0.93	0.78	1.546
VA	1	86	1.04	0.43	1.395
ACMO	1	141	1.12	12~15	1.512
IBOA	1	208	0.98~0.99	2~9	1.476
IDA	1	212	0.88	1~10	1.440~1.442
PHEA	1	192	1.10	5~15	1.518
MBAM	2	154	1.24	Solid	1.488
BDDA	2	198	1.05	8	1.456
HDDA	2	226	1.01~1.03	5~10	1.455~1.457
HDEODA ^+^	2	314	1.01~1.05	10~30	1.461
DEGDA	2	214	1.12	12	1.463
TEGMA	2	286	1.07~1.09	5~30	1.461
TTEGDA	2	302	1.11	5~30	1.465
PEGDA ^+^	2	308~508	1.11~1.12	15~65	1.463~1.467
TPGDA	2	300	1.03	10~15	1.450
PPGDMA ^+^	2	536	1.00~1.01	30~50	1.450
NPGPO2DA	2	328	1.01	10~30	1.440~1.447
BPAE2DMA	2	453	1.12	1800	1.542~1.544
UDMA	2	471	1.11	8500	1.485
TMPTA	3	296	1.11	80~140	1.474
TMPETA ^+^	3	693	1.10~1.11	60~120	1.471
Di-TMPTA	4	467	1.10~1.15	350~800	1.479
EPTTA ^+^	4	550~718	1.14~1.16	100~200	1.475
DPHA	5/6	523~579	1.16	4000~7000	1.488~1.490

^+^ indicates a commercial product that is a mixture of homologues or isomers, rather than a single, pure compound.

**Table 2:** Parameters of the proposed UWB wide-beam antenna.

Parameter	W_d_	L_d_	W_t_	L_t_	L_f_	H_d_
Value (mm)	20.5	27	0.7	22	4	5
Parameter	W_f_	W_s_	L_s_	L_g_	L_gap_	H_s_
Value (mm)	1.9	35.5	43.2	16	0.7	0.762

**Table 3:** Parameters of slotted UWB wide-beam DRA.

Parameter	W_d_	L_d_	W_t_	L_t_	L_f_	H_d_	W_slot_
Value (mm)	20.5	27	0.7	22	4	5	7
Parameter	W_f_	W_s_	L_s_	L_g_	L_gap_	H_s_	L_slot_
Value (mm)	1.9	35.5	43.2	16	0.7	0.762	10

**Table 4:** Comparisons between this work and the state-of-the-art literature.

Ref.	Fabrication Method	Bandwidth (MHz)	Antenna Size (mm^3^)	HPBW (°)		Dielectric Properties		Peak Gain (dB)
				E-Plane	H-Plane	Dielectric Constant	Loss Tangent	
[36]	FDM	4.80–9.94 GHz (69.7%)	0.67λ_0_ × 0.21λ_0_ × 0.54λ_0_	n.a. (~60)	n.a. (~60)	10 and 3	n.a.	8.3
[37]	n.a.	8.88–11.04 GHz (21.7%)	0.77λ_0_ × 0.19λ_0_ × 0.67λ_0_	n.a. (~60)	n.a.	2.2	9 × 10^−4^	4.9
[20]	fused filament fabrication (FFF)	33% (sub 6G) 27% (mm wave)	0.87λ_0_ × 0.87λ_0_ × 0.35λ_0_	32	32	9 and 4	2.9 × 10^−3^	7.2 at 3.2 GHz 18 at 31.5 GHz
[21]	FDM	21–30 GHz (35.29%)	2.2λ_0_ × 2.2λ_0_ × 0.17λ_0_	57	46	10	3 × 10^−3^	11.8
[38]	FFF	5.3–8.8 GHz (49.65%)	0.459λ_0_ × 0.376λ_0_ × 0.411λ_0_	90	60	2.2 and 7.5	1 × 10^−3^	5.9
This work	SLA	2.75–5.75 GHz (70.59%)	0.598λ_0_ × 0.491λ_0_ × 0.069λ_0_	140–210	110–320	9.2	1.6 × 10^−3^	5.5

## Data Availability

The raw data supporting the conclusions of this article will be made available by the authors on reasonable request.
